# Illusory percepts of curvilinear self-motion when moving through crowds

**DOI:** 10.1167/jov.23.14.6

**Published:** 2023-12-19

**Authors:** Anna-Gesina Hülemeier, Markus Lappe

**Affiliations:** 1 Department of Psychology, University of Münster, Münster, Germany; 2 Department of Psychology, University of Münster, Münster, Germany

**Keywords:** optic flow, biological motion, point-light walkers, path perception

## Abstract

Self-motion generates optic flow, a pattern of expanding visual motion. Heading estimation from optic flow analysis is accurate in rigid environments, but it becomes challenging when other human walkers introduce independent motion to the scene. Previous studies showed that heading perception is surprisingly accurate when moving through a crowd of walkers but revealed strong heading biases when either articulation or translation of biological motion were presented in isolation. We hypothesized that these biases resulted from misperceiving the self-motion as curvilinear. Such errors might manifest as opposite biases depending on whether the observer perceived the crowd motion as indication of his/her self-translation or self-rotation. Our study investigated the link between heading biases and illusory path perception. Participants assessed heading and path perception while observing optic flow stimuli with varying walker movements. Self-motion perception was accurate during natural locomotion (articulation and translation), but significant heading biases occurred when walkers only articulated or translated. In this case, participants often reported a curved path of travel. Heading error and curvature pointed in opposite directions. On average, participants perceived the walker motion as evidence for viewpoint rotation leading to curvilinear path percepts.

## Introduction

Observer motion creates a visual pattern known as optic flow (Gibson, 1950). The instantaneous optic flow field, i.e., the optic flow at a given point in time, contains translational and rotational components that directly result from the translation and rotation of the observer's eye. The ability to disentangle translation information from confounding rotations, such as eye rotation or movement along a curve, is crucial for self-motion control (Warren & Hannon, 1988; Royden, Banks, & Crowell, 1992; van den Berg, 1992; Lappe & Rauschecker, 1993; Perrone & Stone, 1994; Beintema & van den Berg, 1998; Lappe, Bremmer, & van den Berg, 1999; Andersen & Saidpour, 2002). Recovering observer translation (instantaneous heading) from an instantaneous flow field is mathematically possible in a rigid environment (Longuet-Higgins & Prazdny, 1980). In a rigid environment, all motion signals in the optic flow field arise from the same six parameters of self-motion (i.e., translation and rotation). Then, the generation of flow from self-motion can be computationally inverted to estimate the heading. Much research has shown that humans can perceive heading under these conditions (e.g., Cutting, 1986; Warren & Hannon, 1988; Cutting, Springer, Braren, & Johnson, 1992; Royden et al., 1992; Warren, Kay, Zosh, Duchon, & Sahuc, 2001; Li, Sweet, & Stone, 2006).

In scenes that violate the assumption of rigidity, for example, if objects move in the scene, heading perception becomes biased by the independent object motion (Warren & Saunders, 1995; Royden & Hildreth, 1996; Layton & Fajen, 2016; Li, Ni, Lappe, Niehorster, & Sun, 2018). A particularly relevant situation occurs when other people move in the scene, such as when a traveling observer encounters another walker or a group of walkers (Riddell & Lappe, 2017; Riddell & Lappe, 2018; Riddell, Li, & Lappe, 2019; Hülemeier & Lappe, 2020; Koerfer & Lappe, 2020; Mayer, Riddell, & Lappe, 2021).

When humans walk, they distinctly articulate their arms and legs. Translation and limb articulation characterize human locomotion, also termed biological motion (Johansson, 1973). The combination of translation and limb articulation in natural locomotion delivers information about the speed and direction of the walker (Giese & Lappe, 2002; Fujimoto & Sato, 2006; Masselink & Lappe, 2015; Thurman & Lu, 2016). Thus, while biological motion walkers violate the rigidity assumption, they also contain information for a remedy of that violation. On the one hand, the biological motion of the walker introduces noise into the optic flow pattern and destroys the rigidity of the scene, thereby causing heading biases (Riddell & Lappe, 2017, Riddell & Lappe, 2018; Riddell et al., 2019; Hülemeier & Lappe, 2020; Koerfer & Lappe, 2020). On the other hand, limb articulation can provide valuable information about the walker's motion (Giese & Lappe, 2002; Masselink & Lappe, 2015), which can be used to reduce the noise and compensate for the independent motion (Riddell & Lappe, 2018; Hülemeier & Lappe, 2020).

**Figure 1.**
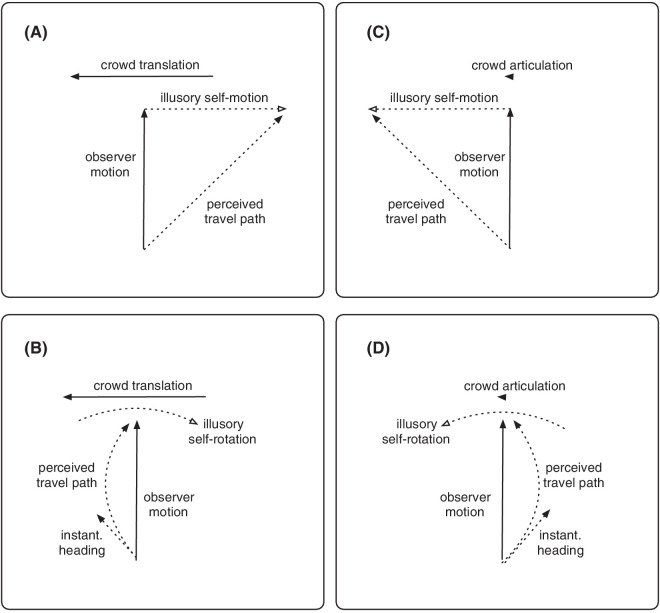
Systematic explanation biases in self-motion perception when the walkers appear as either figure skaters (translation-only condition, left column) or walkers on a treadmill (articulation-only condition, right column). Biases differ depending on whether the observer takes the object's motion as evidence of a translation (upper row) or a rotation of the viewpoint (lower row). Solid lines indicate physical motion and dotted lines denote perceived motion. (**A**) A leftwards translation of the crowd combined with the forward self-motion of the observer indicates a heading to the right (i.e., the opposite facing direction of the crowd). (**B**) Leftward translation of the crowd could alternatively be interpreted as the result of a rightward self-rotation of the observer. This would result in a leftward heading bias (i.e., in the facing direction of the crowd) and a path that curves rightward (i.e., opposite the facing direction of the crowd). (**C**) In the articulation-only condition, the walkers of the crowd articulate their limbs but do not translate. To the observer, this might appear as a self-translation along with the crowd, which, in combination with the observer's forward self-motion induces a perceived heading in facing direction. (**D**) Alternatively, the articulation-in-place may induce the percept of a rotation of the observer's reference frame, which, in combination with the observer's forward self-motion predicts a path percept curved to the left (i.e., in facing direction) and a heading error against facing direction. Note that heading biases and curve directions are opposite between the translation-only and the articulation-only conditions.

Indeed, the combination of limb articulation and walker translation in natural locomotion allows proper heading perception (Riddell & Lappe, 2018; Hülemeier & Lappe, 2020). Conversely, when walkers only move their limbs but do not translate, as if walking on a treadmill, or when walkers only translate through the scene and do not move their limbs, like an ice skater, very large heading errors, up to several tens of degrees, occur (Hülemeier & Lappe, 2020). Such large heading errors indicate a serious misperception of the situation. Notably, the errors exhibited high individual variability, with some participants consistently demonstrating heading errors in the direction of walker movement, while others consistently exhibited errors in the opposite direction. We proposed earlier that these divergent error biases may be attributed to the independent object motion and depend on whether the observer interprets the object motion as evidence of self-translation or self-rotation (Hülemeier & Lappe, 2020). This would be entirely consistent with the motion in the stimulus. For example, if a crowd of walkers translates leftward while the observer moves forward, the leftward translation of the crowd adds a translational component to the flow field that indicates a heading to the right (Figure 1A).

However, there is evidence in the literature that observers sometimes misconstrue sideways translation that is added to a flow field that displays forward self-motion as evidence of self-rotation. For example, an illusory transformation of topic flow fields presented by Duffy and Wurtz (1993) is often explained by a percept of self-rotation in addition to forward self-motion such that the perceived heading is shifted in the direction of the added translation (Duffy & Wurtz, 1993; Lappe & Rauschecker, 1993; Royden & Conti, 2003). Likewise, when objects within a flow field translate independently from the observer, biases in heading estimation can occur that can be explained by a perceived self-rotation of the observer (Royden & Hildreth, 1996; Li et al. 2018). Applied to our stimulus, the leftward translation of the crowd could therefore also be construed as the result of an illusory self-rotation of the observer to the right (Figure 1B). In that case, the situation would be consistent with a leftward heading and possibly a curved path. These different percepts appear possible because the differentiation between straight and curved paths from the flow field is difficult for human observers. Even though the visual system can accurately estimate heading from an instantaneous flow field (Li et al., 2006), the instantaneous flow field alone is insufficient to exhaustively attribute the source of rotation, i.e., whether the rotational component is due to a rotation of the viewpoint (e.g., an eye movement) or the movement along a curved path (Royden, 1994, Royden, Crowell, & Banks, 1994; Li & Warren, 2000; Royden, Cahill, & Conti, 2006). The differences between these two possibilities are revealed only by the evolution of the flow field over time and become prominent when combined with depth information from motion-parallax or a reference object from the environment (Stone & Perrone, 1997; Li & Warren, 2000). Observers often confuse rotations of the viewpoint with movements along a curved path. For example, Bertin, Israël, & Lappe (2000) simulated self-motion on straight or circular paths together with viewpoint rotations and examined under which circumstances participants misperceived the curvature of their traveled path. Although participants demonstrated an accurate perception of viewpoint rotation (yaw), their heading perception remained fixed relative to the trajectory or space, resulting in the misplacement of their heading and subsequent misperception of the trajectory. Consequently, participants attributed eye rotation to a rotation of the path, leading to erroneous trajectory perception. The degree to which observers misrepresent their travel path depends on the combination of gaze and path rotation (Bertin et al., 2000). Cheng and Li (2012) showed that individuals perceive curved paths of travel through the rotation in the retinal velocity field. Path perception is most successful when the simulated observer gaze direction is aligned with the instantaneous heading direction (Li & Cheng, 2011) as if the visual system presumes a constant heading with respect to the body while traveling on a curved path.

To summarize, the translation-only stimulus might produce two ambiguous percepts, either a self-translation toward a heading that is displaced against the direction of translation and facing of the crowd (Figure 1A), or a movement along a curved path with an instantaneous heading toward the direction of facing of the crowd (Figure 1B).

Similar considerations may explain the biases observed in the condition in which the walkers only articulate but do not translate. When viewing a point-light walker, observers can derive information about the walker's translation from limb articulation (Masselink & Lappe, 2015). If the walker remains stationary and does not translate the limb articulation induces an illusory movement of the environment called the back scroll illusion (Fujimoto, 2003; Fujimoto & Sato, 2006; Fujimoto & Yagi, 2008). Importantly, this illusory background motion is directed in the opposite facing and walking direction of the walker. The illusory motion of the environment could shift the reference frame and might result in a perceived self-motion of the observer, particularly when combined with forward self-motion (Hülemeier & Lappe, 2020). In that case, there are again two possibilities for heading biases, as depicted in Figures 1C and D. Either observer could experience a self-translation along with the crowd (i.e., a self-translation that keeps the view of the crowd fixed as the observer moves with the crowd). Note that this would be consistent with the background/environment motion opposite the facing direction of the crowd. This percept should induce a perceived heading in the facing direction of the crowd (Figure 1C). Alternatively, the observer experiences a self-rotation (Figure 1D) that keeps the view of the crowd fixed. This self-rotation would be in the facing direction of the crowd, which should result in a curved path percept and a heading error against the facing direction of the crowd (Figure 1D). To summarize, heading biases and curved path percepts might be stimulated by the induced illusory motion from limb articulation similar to the translation in Figure 1A and B, but heading biases and curve directions would be opposite between the translation-only and the articulation-only conditions because the induced motion from the articulation-only condition is opposite to the translation in the translation-only condition.

In the present study, we search for evidence that the heading biases reported earlier for combinations of optic flow and biological motion are related to illusory path perception (Figure 1). Across a series of experiments, we asked participants to assess their perceived heading direction and path. In these experiments, we present optic flow stimuli that simulate the movement of an observer relative to a group of walkers that could either walk naturally with a proper combination of articulation and translation, articulate without translation as if walking in place, or translate without articulation like an ice skater. In the fourth condition, the walkers simply stand in place forming a rigid environment. We expect from earlier results (Hülemeier & Lappe, 2020) that heading errors will be small in the latter condition as well as in the natural locomotion condition but large in the articulation-only and the translation-only conditions. In these conditions, according to our above hypothesis (Figure 1), we expect participants to indicate a curved path of travel as a result of an illusory rotational component and a misattribution of this component to a curvature of the travel path. We further expect that heading error and curvature direction are inversely related to each other and that errors in articulation-only conditions are opposite to errors in the translation-only condition.

Furthermore, we also varied the information about the scene and scene depth that is available to the observer to separate translational and rotation components of the flow field. In one condition, all walkers of the group have the same distance from the observer such that the group contains no-motion-parallax. In a second condition, the walkers have different distances from the observer, giving rise to motion-parallax (Hanes, Keller, & McCollum, 2008). Motion-parallax is important to separate translation and rotation in the instantaneous flow field and, thus, might influence the misperception of a rotational component (Koenderink & van Doorn, 1987; Warren & Hannon, 1990; Lappe & Rauschecker, 1993). In the third condition, we further added a ground plane to the scene. The ground plane contains veridical optic flow related to observer motion. Moreover, the ground plane provides a reference by which translation of the walkers, or walking-in-place, respectively, could be estimated. Thus we expect this condition to reduce misperception of self-motion.

## Methods

Our study comprised three experiments. Because of similar experimental setups and research questions, we combine the first and second experiments for our analysis.

### Sample

We recruited 24 observers (female: n = 15, male: n = 9; age ranged from 18 to 34 years: *M* = 22.83, *SD* = 4.03) to join the first experiment, and 30 volunteers to participate in our second experiment (female: n = 22, male: n = 8; age ranged between 18 and 29: *M* = 21.73, *SD* = 2.82) from the University of Münster. All participants had normal or corrected-to-normal visual acuity and were unaware of the study's objectives. Participation was voluntary, anonymous, and compensated by either course credits or money. The study was approved by the Ethics Committee of the Department of Psychology and Sport Science at the University of Münster.

### Experimental settings

The experiment took place in a darkened laboratory room. Stimuli were generated in MATLAB (version R2020b, The MathWorks, Natick, MA) with the OpenGL libraries add-ons of Psychtoolbox (version 3.0.17, Kleiner, Brainard, & Pelli, 2007). We connected an Apple MacBook Pro (equipped with a Radeon Pro 560 × 4GB graphic card) to a VDC Display Systems Marquee 8500 projector to project the stimuli onto a 250 cm × 200 cm backlit screen. The screen resolution was 1024 × 768 pixels with a frame rate of 60 Hz. During the stimulus presentation, subjects sat about 100 cm away from the screen. Their visual field had a size of 102° × 90°. Responses were collected using a computer mouse.

### Walker creation

We created a crowd of eight life-sized point-light walkers (Johansson, 1973) from the motion-tracking data of a walking person (de Lussanet et al., 2008). Each walker consisted of 12 white points whose positions corresponded to human joints (shoulders, elbows, hips, wrists, knees, and ankles). The walkers faced either collectively to the left (−90°) or right (90°). Each walker started individually with a random starting position in the gait cycle.

### Self-motion simulation

We simulated the participant's self-motion along a straight path at 0.8 m/s. Self-motion and walker translation velocities were equal. Heading varied within 12° from the screen center.

### Experimental scenes

**Figure 2.**
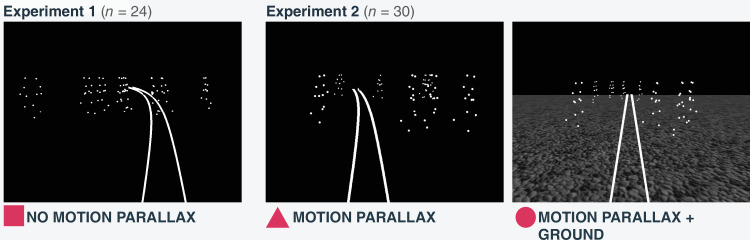
Stimuli including report procedure. Participants’ self-motion toward a crowd of eight point-light walkers was simulated. The no-motion-parallax scene presents the crowd standing at a similar depth position, containing no cues of motion parallax. The motion-parallax scene presents half of the crowd further away and half of the crowd at the same distance as in the no-motion parallax condition. This placement induces motion parallax cues. The ground scene adds a gray gravel ground to the scene. The walkers are placed the same as in the motion-parallax condition. The white path shows the self-motion reporting procedure. Participants sketched their perceived path to their perceived heading by adjusting the curvature, curvature direction, and position of the visual path indicator. The plots in the results section employ geometric symbols as references to the respective scene.

We tested three (experiment 1: one scene, experiment 2; two scenes) experimental scenes successively comprising more depth information (Figure 2). In each case, the simulated virtual world spanned over 20 m scene depth. The first scene showed only the walkers, which were positioned between 7 m and 9 m. We refer to this scene as the “no-motion-parallax scene.” The second scene placed half of the walkers between 7 and 9 m in depth (like in the first experiment), and the other half twice as far away (i.e., 14 to 18 m). Their size was scaled with depth. The scene thus contains stronger motion-parallax cues, and we refer to it as the “motion-parallax scene.” The third scene added a gray-textured ground plane. We call this experimental scene the “ground scene.” The crowd in the ground scene was positioned in the same way as in the motion-parallax scene.

### Walker conditions

To explore the influence of biological motion components on heading perception from optic flow analysis, we designed four conditions: static, natural locomotion, translation-only, and articulation-only. In the static condition, the walkers resembled static figures, maintaining a fixed position. The natural locomotion condition presented the walkers naturally moving through the world while articulating their limbs. This condition combined both translation and articulation of biological motion. In the translation-only condition, walkers slid through the scene without any limb movement, resembling figure skaters moving in the direction they faced. Conversely, the articulation-only condition depicted walkers moving their limbs without physical translation, as if on a treadmill.

### Procedure

Participants saw a trial simulating their self-motion towards a crowd, and afterwards used a mouse to report their heading direction and adjusted a visual path indicator (Figure 2) to their perception of their self-motion. Heading was controlled by horizontal mouse movements, while vertical mouse movements adjusted the curvature. Upward movements straightened the path, while downward movements curved it. Pressing the right mouse button inverted the curvature direction of the path indicator, which was chosen randomly in each trial. Subjects registered their response by pressing the left mouse button.

Following the instruction, participants completed a practice block without data collection and performance feedback. The practice block covered two randomized repetitions of all stimulus combinations. Once the practice block was finished, data collection started. An experimental block contained 20 repetitions of all stimulus combinations in a randomized order. In Experiment 1, each participant completed a total of 320 trials (4 walker conditions × 2 facing directions × 20 repetitions × 2 blocks × 1 scene) equally distributed across two experimental blocks. In experiment two, each participant completed a total of 640 trials (4 walker conditions × 2 facing directions × 20 repetitions × 2 blocks × 2 scenes). A trial lasted about 2500 ms. The entire experiment, including a short break between the experimental blocks, took about half an hour for Experiment 1 and about an hour for Experiment 2.

### Data preparation

Our analysis examined the relationship between curvature and heading error. We calculated the instantaneous heading error as the difference between the estimated and actual heading in degree of visual angle. The curvature is reported in m^−^^1^. For analysis purposes, we recoded the instantaneous heading error and curvature in the direction in which the walkers faced, with positive values indicating the same direction as the facing of the crowd, and negative values in the direction opposite to the facing of the crowd. Note that the crowd, when translating, always translated in the facing direction.

### Analysis procedure

We analyzed the curvature and heading estimates per condition and scene. Depending on the data distribution, we applied either parametric or non-parametric tests. Depending on the chosen test, we report and plot either the mean for the parametric test or the median for the non-parametric one.

Across conditions and scenes, we assessed whether participants perceived their path as linear employing frequentist parametric Student's *t*-test or non-parametric Wilcoxon rank-sum-tests. If these tests did not reach significance, we complemented the analysis with Bayesian statistics (Wagenmakers et al., 2018) to look for evidence in favor of the null hypothesis (i.e., a linear path percept). The Bayesian *t*-test generates a Bayes factor known as BF_10_. BF_10_ values <1 support the null hypothesis, signifying no observable difference, whereas values exceeding 1 favor the alternative hypothesis, implying a distinction between conditions. The greater the deviation from 1 in BF_10_ values, the stronger the evidence becomes. BF_10_ values falling between 0.33 and 3 are generally considered inconclusive (Jeffreys, 1961).

In addition, we used the linear mixed model framework for two reasons. First, the dependent variables in our study exhibited non-normal distributions across conditions, violating the assumption of analysis of variance (ANOVA) based on ordinary least squares regression models. Linear mixed models can accommodate non-normal data, providing more robust estimates (Arnau, Bono, Blanca, & Bendayan, 2012). Second, the mixed-modeling framework offers greater flexibility, accuracy, and power for repeated-measures data by accounting for both fixed and random effects, as well as accommodating varying variances, covariances, and distributions (Kristensen & Hansen, 2004; Jaeger, 2008).

We fitted linear mixed models using restricted maximum likelihood criterion and nloptwrap optimizer with random intercept and constant slope for participants. Our model predicted the instantaneous heading error or curvature (both in the walker-facing direction) by the experimental conditions. The model included participant id as a random effect and conditions as fixed effects with four levels. We assumed that curvature and heading error exhibited some residual variation associated with participants due to the data structure. Due to the data structure, it was impossible to further cluster the observations by random effects. We obtained standardized parameters by fitting the model on a standardized version of the dataset. The 95% confidence intervals (CI) and *p*-values were aligned to the Wald approximation. Effect sizes were labeled following Field's (2013) recommendations. Significant effects were followed by a post hoc analysis to decisively test differences between conditions with *p* value adjustment according to Holm (1979).

## Results

We aimed to understand how biological motion components, encompassing translation and limb articulation, interplay with illusory path perception and heading biases. To describe the results and provide a coherent approach to our research question we employed a six-step analytical procedure. Initially, we assessed whether participants could accurately perceive their heading direction and path of self-motion. For this purpose, we first provided detailed descriptive statistics and combined them with inferential analyses. The findings are organized by condition starting with section 1 about the static and natural locomotion conditions, and then proceeding with section 2 about the translation-only and articulation-only conditions. In both sections, we present findings on scene level for each condition. Based on the baseline assessment, we explored the interaction between the walker condition and the scene on the perceived self-motion trajectory (section 3). After this detailed analysis of the curvature, we continued with the analyses of the heading error and investigated how scene cues improved heading estimation precision (sections 4 and 5). Concluding our analysis, we assessed the relationship between perceived curvature and perceived heading direction (section 6).

### Participants correctly perceived linear self-motion in the static and natural walking conditions

#### Descriptive findings of the static condition

The static condition, in which independent object motion was absent, provided a baseline for heading estimation from optic flow analysis. As expected, the average instantaneous heading error was around 0° across scenes, so within the range of accurate locomotion (Cutting, 1986) (no-motion-parallax scene: facing −90: *M* = −1.52, *SD* = 10.94; facing 90: *M* = −0.25, *SD* = 10.95; motion-parallax scene: facing −90: *M* = −2.74, *SD* = 7.99; facing 90: *M* = −2.23, *SD* = 7.31; ground scene: facing −90: *M* = −2.96, *SD* = 7.68; facing 90: *M* = −3.20, *SD* = 7.90). The average estimates of path curvature across scenes and facing directions were at 0.00, indicating a straight path, also as expected. The standard deviations of the curvature reports decreased from the first scene (facing −90: *SD* = 0.05, facing 90: *SD* = 0.04) to the second (facing −90: *SD* = 0.03, facing 90: *SD* = 0.03) and to the third scene (facing −90: *SD* = 0.02, facing 90: *SD* = 0.02), consistent with the increase of cues that allowed separate translation and rotation in the optic flow. These results suggest that participants could perceive their path accurately in the static condition.

#### Inferential analyses on path perception in the static and natural locomotion conditions

**Figure 3.**
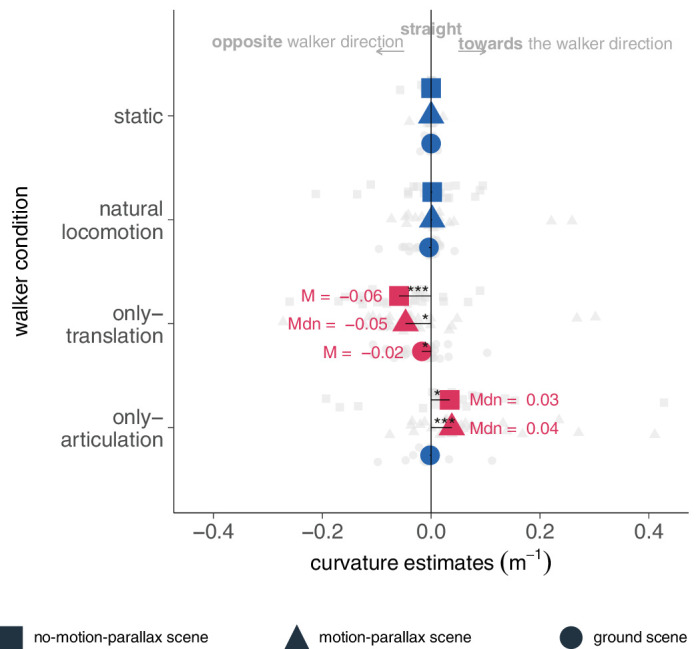
Curvature estimates per conditions and scene. Negative values denote curvature estimates in the opposite direction to the walker-facing direction, while positive ones denote curvature estimates toward the walker-facing direction. The gray data points represent the participant's average curvature in facing estimates. The colored data points represent average curvature estimates. Red points indicate estimates differed significantly from zero whereas the blue ones denote nonsignificant curvature estimates. The shape of the points symbolizes the experimental scenes.

For statistical analysis, we tested whether the average curvature estimates differed significantly from zero. Our analysis revealed a distinct pattern of curvature magnitude and direction depending on the walker condition (Figure 3). In the static and natural locomotion conditions of the no-motion-parallax scene, curvature estimates did not differ significantly from zero (static: *Mdn* = 0.000, *SD* = 0.01, 95% CI [−0.01, 0.01], *W* = 84, *p* = 0.739, *r* (rank biserial) = 0.15, 95% CI [0.01, 0.58]; natural locomotion: *Mdn* = 0.002, *SD* = 0.07, 95% CI [−0.02, 0.02], *W* = 155, *p* = 0.897, *r* (rank biserial) = 0.03, 95% CI [0.01, 0.48]). The Bayesian *t*-test provides moderate evidence for the null hypothesis signifying participants perceived their path as linear in both the static and natural locomotion conditions (static: BF_10_ = 0.294% ± 0.03%; natural locomotion: BF_10_ = 0.242% ± 0.02%).

Put differently, participants perceived their path of self-motion correctly as linear. This accurate self-motion perception was consistent across the other scenes (motion-parallax scenes: static: *Mdn* = 0.000, *SD* = 0.01, 95% CI [−0.002, 0.002], *W* = 127, *p* > 0.999, *r* (rank biserial) = 0.00, 95% CI [−0.44, 0.45], BF_10_ = 0.209% ± 0.03%; natural locomotion: *Mdn* = 0.002, *SD* = 0.07, 95% CI [−0.01, 0.01], *W* = 245, *p* = 0.559, *r* (rank biserial) = 0.13, 95% CI [−0.28, 0.50], BF_10_ = 0.306% ± 0.03%; ground-scene: static: *Mdn* = 0.000, *SD* = 0.01, 95% CI [−0.002, 0.001], *W* = 150, *p* = 0.515, *r* (rank biserial) = −0.15, 95% CI [−0.53, 0.28], BF_10_ = 0.249% ± 0.03%; natural locomotion: *Mdn* = −0.004, *SD* = 0.03, 95% CI [−0.016, 0.001], *W* = 148, *p* = 0.084, *r* (rank biserial) = −0.36, 95% CI [−0.66, 0.03], BF_10_ = 1.132% ± 0.02%). Correct path perception was accompanied by an increased precision from the motion-parallax to the ground scene, shown by significantly decreased variance (one-tailed testing; natural locomotion: *F*(29, 29) = 6.41, *p* < 0.001). The decreased variance additionally suggests that the visual system processed the independent optic flow from the ground in the ground scene to increase the precision of self-motion perception. Linear path percepts in the static condition demonstrate that participants were capable of perceiving their self-motion correctly and accurately. Furthermore, these results indicate that visual perception of self-motion is robust to the presence of naturally translating biological motion that disturbs the optic flow field pattern.

### Participants misperceived their self-motion as curvilinear in the translation-only and articulation-only conditions

#### Descriptive findings of the translation-only condition

In the translation-only condition in the no-motion-parallax scene, our study revealed a negative and statistically significant mean curvature in opposite facing direction (*M* = −0.06, *SD* = 0.08, 95% CI [−0.09, −0.03], *t*(23) = −3.65, *p* = 0.001) with a moderate effect size (Cohen's *d* = −0.74, 95% CI [−1.22, −0.29]). Despite the introduction of motion-parallax and ground plane, we did not observe a reduction in the curvature percept (motion-parallax scene: *Mdn* = −0.05, *SD* = 0.10, 95% CI [−0.07, −0.02], *W* = 87, *p* = 0.003, *r* (rank biserial) = −.63, 95% CI [−.82, −.31]; ground scene: *M* = −0.02, *SD* = 0.04, 95% CI [−0.03, −0.001], *t*(29) = −2.19, *p* = 0.036, Cohen's *d* = −0.40, 95% CI [−0.78, −0.03]). These significant curvature estimates demonstrate the combination of the ground and motion-parallax could not resolve the path ambiguity, leading to a misperception of self-motion along a curvilinear, instead of a linear, path. At least the ground plane compared to motion-parallax alone reduced the variance (one-tailed testing, *F*(29, 29) = 6.03, *p* < 0.001) so that the test subjects became more precise in their assessment. The optic flow from the ground was thus processed, but it did not help to correct the path percept.

#### Inferential analyses on path perception in the and articulation-only condition

For the articulation-only condition, our study revealed a dependence of self-motion perception on scenes (Figure 3). For the no-motion-parallax scene, we found a significant median curvature in facing direction (*Mdn* = 0.034, *SD* = 0.12, 95% CI [0.01, 0.06], *W* = 230, *p* = 0.022) with a large effect size (*r* (rank biserial) = 0.54, 95% CI [0.14, 0.78]). The introduction of motion-parallax did not resolve the erroneous curvature perception, as evidenced by the significant differences from zero observed in the curvature estimates in the motion-parallax scene (*Mdn* = 0.038, *SD* = 0.10, 95% CI [0.03, 0.09], *W* = 404, *p* < 0.001, *r* (rank biserial) = 0.74, 95% CI [0.49, 0.88]). This suggests a misperception of self-motion along a curvilinear path instead of the true straight path. In the ground scene, the estimated curvature became smaller and was no longer different from zero (*Mdn* = −0.002, *SD* = 0.03, 95% CI [−0.01, 0.00], *W* = 103, *p* = 0.067, *r* (rank biserial) = −0.41, 95% CI [−0.71, 0.00], BF_10_ = 0.230% ± 0.03%), indicating that participants perceived their self-motion more correctly along a linear path. The variance of curvature estimates also decreased, implying improved precision of self-motion estimation due to the use of independent optic flow from the ground (one-tailed testing, *F*(29, 29) = 10.79, *p* < 0.001).

In conclusion, the scene-wise analysis indicated that the curvature estimates in the articulation-only condition were significantly larger than zero in the motion-parallax scene, whereas in the ground scene, the curvature estimates diminished and did not differ significantly from zero. This result pattern provides evidence for a change in curvature percepts across different scenes, highlighting the complexity of visual processing involved in the self-motion perception from biological motion.

### The interaction of biological motion and scene cues is crucial for correct self-motion path perception

In the next analysis, we sought a coherent interpretation of the interaction between walker condition and scene. We calculated a linear mixed model predicting the curvature estimates in facing direction by the walker conditions, and for the second experiment, by scene. Note that we calculated separate models for the experiments (experiment 1: no-motion-parallax scene; experiment 2: motion-parallax scene, ground scene) because of the structure of data acquisition.

In the first experiment, the main effect of the condition was statistically significant and large (*F*(3) = 7.94, *p* < 0.001, η^2^ (partial) = 0.26, 95% CI [0.10, 1.00]). Post hoc analyses revealed that the translation-only condition was significantly different from all other conditions (*p* < 0.05). For the second linear model, we included an interaction term between condition and ground, due to the within-subject design. The ANOVA based on the linear mixed model showed that both main effects of the condition (*F*(3) = 11.39, *p* < 0.001, η^2^ (partial) = 0.14, 95% CI [0.07, 1.00]) and ground (*F*(1) = 7.72, *p* = 0.006, η^2^ (partial) = 0.04, 95% CI [0.01, 1.00]) were statistically significant, as well as their interaction (*F*(3) = 7.37, *p* < 0.001, η^2^ (partial) = 0.10, 95% CI [0.04, 1.00]). The post hoc analyses confirmed significant differences between most conditions (*p* < 0.026; static vs. natural locomotion *p* = 0.841) and larger curvature estimates in the motion-parallax than the ground scene (*M_diff_* = 0.019, *p* = 0.006).

The interaction effect of the scene and walker condition was further examined by comparing the curvature estimates within conditions between scenes. Only the curvature in the articulation-only condition changed significantly between scenes (*M_diff_* = 0.06, *p* < 0.001; see Figure 3). This shift suggests that curvature percepts may change if sufficient environmental cues are available. The non-significant changes within the static (*p* = 0.999) and natural locomotion conditions (*p* = 0.999) are reasonable, because the descriptively low curvature estimates indicated a correct linear path percept. In the translation-only condition, there were no significant differences between scenes, suggesting that the increased cues did not change path perception.

### Scene depth cues improve heading precision

#### Heading error descriptive analysis

**Figure 4.**
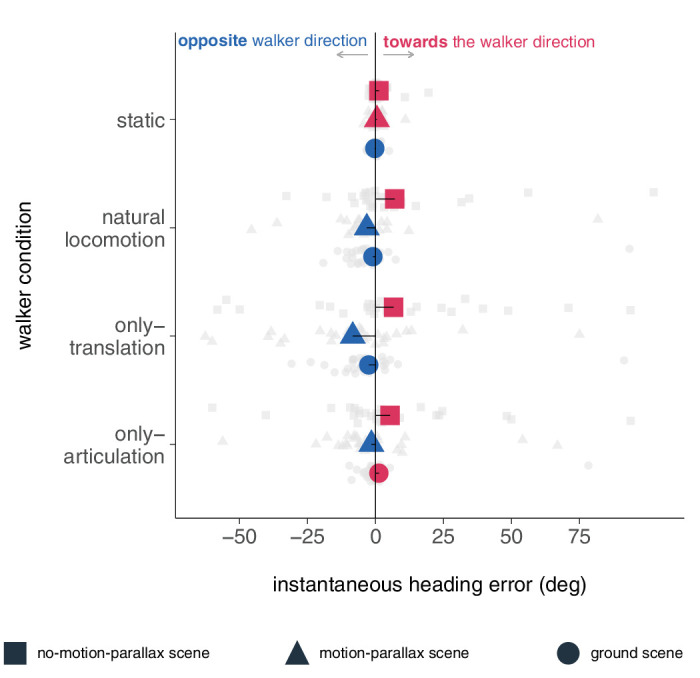
Instantaneous heading error per conditions and scene. The gray data points represent the participant's average instantaneous heading error in facing direction. The colored data points represent average instantaneous heading errors. The shape of the points symbolizes the experimental scenes. Negative values (blue) denote instantaneous heading errors in opposite direction to the walker-facing direction, whereas positive ones (red) denote errors toward the walker-facing direction.

We performed a descriptive analysis to obtain an overview of the distribution of the data and to identify patterns and trends in the data. The results revealed variations in the mean instantaneous heading error across conditions and scenes (Figure 4). In the static condition, the mean instantaneous heading error was 1.27 (*SD* = 4.76) for the no-motion-parallax scene, 0.51 (*SD* = 2.64) for the motion-parallax scene, and −0.25 (*SD* = 1.61) for the ground scene. In the natural locomotion condition, the mean instantaneous heading error successively decreased in magnitude and variance from *M* = 7.09 (*SD* = 26.84) for the no-motion-parallax scene, to *M* = −3.20 (*SD* = 19.31) for the motion-parallax scene, to *M* = −0.96 (*SD* = 18.64) for the ground scene. For the translation-only condition, the mean instantaneous heading error was positive *M* = 6.64 (*SD* = 35.74) for the no-motion-parallax scene, −8.36 (*SD* = 25.92) for the motion-parallax scene, and −2.48 (*SD* = 19.64) for the ground scene. In the articulation-only condition, the mean instantaneous heading error was *M* = 5.36 (*SD* = 30.07) for the no-motion-parallax scene, *M* = −1.50 (*SD* = 20.88) for the motion-parallax scene, and *M* = 1.24 (*SD* = 14.87) for the ground scene.

We evaluated the effect of motion-parallax and ground cues on the perception of locomotion using an *F*-Test to compare the variance between scenes. Our results showed a significant decrease in variance in the static and articulation-only conditions (static: *F*(29, 29) = 2.71, *p* = 0.005; articulation-only: *F*(29, 29) = 1.97, *p* = 0.036). In other words, the ground cues further improved precision of heading perception in the static condition. This increased precision attests to the visual system utilizing additional environmental cues to further improve heading estimation. Furthermore, when only biological motion was available (articulation-only condition), the visual system used the independent optic flow from the scene to improve heading estimation precision. However, the same scene information did not alter the heading estimation precision in the natural locomotion translation-only conditions as the error variance did not show a significant difference (natural locomotion: *F*(29, 29) = 1.07, *p* = 0.425; translation-only: *F*(29, 29) = 1.74, *p* = 0.071).

The large variability in the data (Figure 4), especially in the no-motion-parallax scene, points to individual idiosyncratic differences in heading perception. To further investigate the influence of the condition and scene on heading perception, we next calculated a linear mixed model.

### Idiosyncratic errors either in or opposite the facing direction

#### Inferential analysis

Analogously to the curvature analysis, we calculated a linear mixed model to predict the instantaneous heading error in the facing direction, based on the conditions of the walker. In the first experiment, the main effect of the condition was not found to be statistically significant, with a small effect size (*F*(3) = 0.38, *p* = 0.766, η^2^ (partial) = 0.02, 95% CI [0.00, 1.00]). For the second linear model, we incorporated an interaction term between the condition and ground, owing to the within-subject design. The ANOVA revealed that the main effect of the condition was statistically significant, yet small in magnitude (*F*(3) = 2.83, *p* = 0.040, η^2^ (partial) = 0.04, 95% CI [0.001, 1.00]). Notably, none of the post hoc comparisons between conditions reached significance (*p* > 0.06 Holm-corrected). Neither the main effect of the scene (*F*(1) = 2.76, *p* = 0.098, η^2^ (partial) = 0.01, 95% CI [0.00, 1.00]) nor the interaction effect attained significance (*F*(3) = 0.80, *p* = 0.497, η^2^ (partial) = 0.01, 95% CI [0.00, 1.00]). Together, these findings suggest that the walker condition had a small and limited effect on the instantaneous heading error. We assume that the variability between subjects statistically masks the effect of the condition.

### Relationship between heading error and curvature suggests opposing behavior

**Figure 5.**
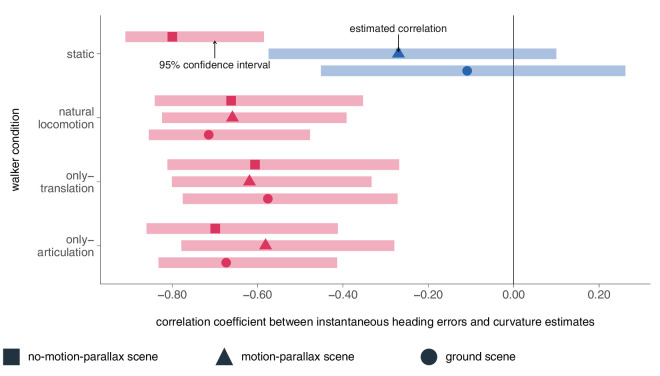
Correlation coefficients including 95% confidence intervals between instantaneous heading errors and curvature estimates per condition and experimental scene (symbols). Red data points indicate significant correlations, whereas blue ones reflect nonsignificant correlations.

We investigated the relationship between curvature and heading error in facing direction across walker conditions and scenes. Figure 5 illustrates the mean curvature in facing direction for each participant in relation to their mean heading error in facing direction. Each subplot represents an experimental condition. The results indicate a strong negative correlation between heading error and curvature, which is statistically significant in nearly all conditions except for the static condition in the motion-parallax and ground scenes (Table 1). Although the confidence intervals for the effect sizes are relatively wide, the magnitude of the effect sizes is considerable.

**Table 1.** Correlation between heading error and curvature estimate per condition and scene.

Scene	Condition	*r*	95% CI	*t*-value	*p* value
No motion parallax	Static	−0.80	[−0.91, −0.59]	*t*(22) = −6.25	<0.001
	Natural locomotion	−0.66	[−0.84, −0.35]	*t*(22) = −4.14	<0.001
	Translation-only	−0.61	[−0.81, −0.27]	*t*(22) = −3.57	0.002
	Articulation-only	−0.70	[−0.86, −0.41]	*t*(22) = −4.59	0.001
Motion parallax scene	Static	−0.27	[−0.57, 0.10]	*t*(28) = −0.58	0.149
	Natural locomotion	−0.66	[−0.82, −0.39]	*t*(28) = −4.63	<0.001
	Translation-only	−0.62	[−0.80, −0.33]	*t*(28) = −4.17	<0.001
	Articulation-only	−0.58	[−0.78, −0.28]	*t*(28) = −3.78	<0.001
Ground scene	Static	−0.11	[−0.45, −0.26]	*t*(28) = −0.58	0.567
	Natural locomotion	−0.71	[−0.85, −0.48]	*t*(28) = −5.40	<0.001
	Translation-only	−0.58	[−0.78, −0.27]	*t*(28) = −3.73	<0.001
	Articulation-only	−0.67	[−0.83, −0.41]	*t*(28) = −4.82	<0.001

The negative correlation suggests an opposite relationship between perceived heading and curvature. For example, when participants produced a heading error in the direction in which the walkers were moving (e.g., to the left), they drew their curvature in the opposite direction (to the right). The significant correlation confirms previous observations (Hülemeier & Lappe, 2020) and considerations (Figure 1) about the instantaneous heading error, and perceived curvature (Bertin et al., 2000; Royden et al., 2006; Wilkie & Wann, 2006).

## Discussion

We aimed to understand how the components of biological motion (translation and limb articulation) are related to illusory path perception (Figure 1) and heading biases. Biological motion is particularly interesting because it can both improve and impede self-motion perception. Combining limb articulation and walker translation in natural locomotion facilitates accurate heading perception (Riddell & Lappe, 2018; Hülemeier & Lappe, 2020), suggesting a balance between the two components of biological motion. However, when walkers only perform limb articulation without translation or only translate without limb movement, significant heading errors occur (Hülemeier & Lappe, 2020), indicating a severe misperception of the situation. The magnitude and direction of these errors varied among individuals and may depend on how the observer might interpret the object motion as either self-translation or self-rotation within the flow field.

### Binary judgment experiment

Our previous experiments have shown that the perceived path of self-motion varies depending on the presence and type of visual cues in the environment. We aimed to provide further evidence by confirming the perceived curvilinear path in the translation-only and articulation-only conditions in a two-alternative forced choice paradigm, in which participants were presented with the motion-parallax scene and asked to select whether their path of self-motion was either linear or curvilinear. The motion-parallax scene was chosen because it provides sufficient ambiguity to perceive the path as curvilinear in the articulation-only and translation-only conditions. Note that self-motion simulation was always along a linear path.

### Methods

#### Sample

We recruited 35 participants from the University of Münster for the third experiment (29 female, five male, one diverse), with ages ranging from 18 to 29 (*M* = 20.57, *SD* = 2.16). Participation conditions remained unchanged from the previous experiments.

#### Experimental setting

We maintained the setting, the motion-parallax scene, and the four walker conditions (static, natural locomotion, translation-only, and articulation-only) from the second experiment.

#### Procedure

The experimental procedure was equivalent to the prior experiment, including the number of stimulus repetitions. By clicking the left or right mouse button, participants determined if the self-motion path was linear or curvilinear. If they chose curvilinear, they additionally indicated the curvature direction (left or right). The entire experiment, including breaks, took about 30 minutes.

#### Data analysis

To assess whether the observed distribution of curvilinear vs. linear responses aligned with what would be expected if participants could perceive their path as linear, we conducted a χ^2^ goodness-of-fit test. Because of the way the test works the counted events must not be equal to zero and must be greater than five. We assumed that in each condition, 10 out of 2800 trials (0.357%) would produce a curvilinear response. As a follow-up analysis, we examined differences in the distribution of the responses (linear vs. curvilinear) across conditions using a χ^2^ test for homogeneity. For pairwise comparisons between conditions, we applied a proportion test with Holm-correction (Holm, 1979) for multiple testing.

### Results and discussion

**Figure 6.**
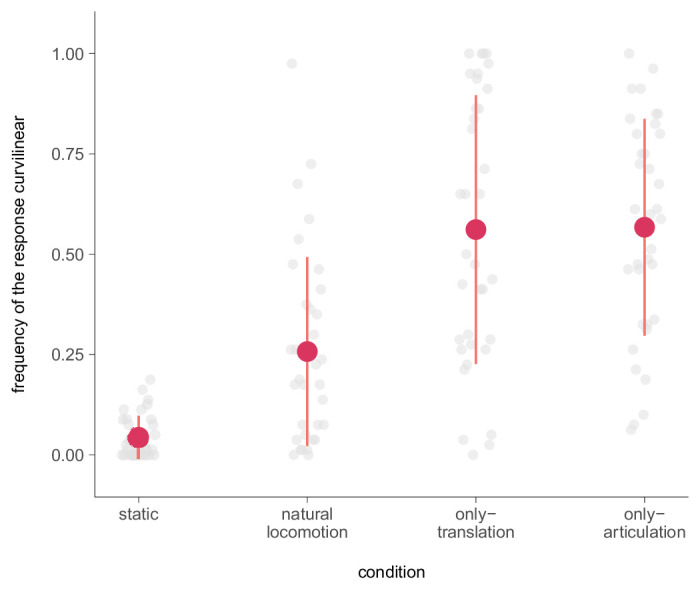
Relative frequency of the answer “curvilinear” per condition. The data points per participant are colored in gray. The red dots represent the mean frequency across all subjects ±1 *SD*.

We calculated the mean relative frequency of the “curvilinear” response for each participant and condition (Figure 6). The results indicate that the frequency of the curvilinear response increased progressively from the static (*M* = 0.04, *SD* = 0.05) to natural locomotion (*M* = 0.26, *SD* = 0.24) to translation-only (*M* = 0.56, *SD* = 0.34) and articulation-only conditions (*M* = 0.57, *SD* = 0.27). Across conditions, participants reported a higher frequency of perceiving their path as curvilinear rather than linear, which was significantly different from the expected frequency (χ^2^(7) = 546775, *p* < 0.001). This increase depending on condition was found to be significant (χ^2^(3) = 2362.2, *p* < 0.001) with a medium effect size (Cohens’ ω = 0.46). Pairwise comparisons revealed significant differences in the frequency of the curvilinear responses (*p* < 0.001) between all conditions except for the comparison between the translation-only and articulation-only conditions, which did not yield a significant difference (*p* = 0.686).

**Figure 7.**
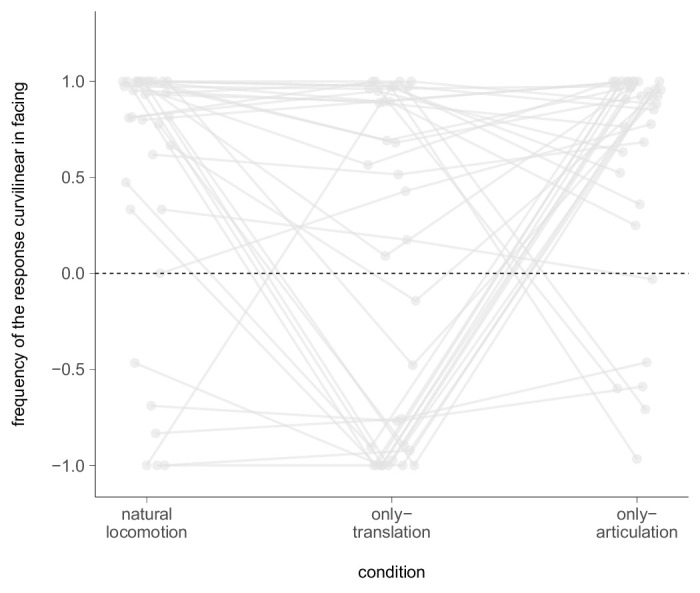
Relative frequency of the curvature in facing judgments (positive values) per condition. Negative values denote judgments in opposite walker-facing direction. Frequency of zero (dotted horizontal line) indicates that 50% of curvature replies were in facing, whereas the remaining 50% were in opposite facing direction. A subject's data points are linked across conditions. Consider we excluded data from participants who did not indicate any curvilinear response.

Next, we investigated whether participants reported curvilinear paths in either a walker-facing or opposite walker-facing direction in the presence of biological motion. Through a careful screening of individual data (Figure 7), we identified distinct biases that were idiosyncratic and direction-dependent. Notably, these idiosyncratic biases canceled out when averaged across participants. Qualitatively, our results suggest a preference for curvilinear paths in the facing direction in the natural locomotion and articulation-only conditions, whereas the opposite direction was preferred during translation-only conditions. In other words, opposing curve perception was observed in articulation-only and translation-only conditions. It is important to note that we excluded data from participants who did not indicate a curvilinear response, meaning the sum of data points to which the frequency refers varies across individuals and conditions. Importantly, our findings are consistent with the analysis of prior experiments (Figure 3), suggesting that our results are robust and generalizable.

The a two-alternative forced choice paradigm used in this study is sensitive to small, continuous changes in path perception because participants must categorically decide instead of gradually bending the path. We found frequent reporting of the perceived path as curvilinear instead of linear in each condition. Assuming a perfectly accurate perception of self-motion irrespective of the walker condition, participants showed a higher frequency of curvilinear responses than expected. Still, they were able to satisfactorily complete the task with only 4% of falsely interpreted judgments in the static condition. The highest frequency of curvilinear responses was found in the translation-only and articulation-only conditions, providing further support for our previous observations that participants indeed perceived their path as curvilinear rather than falsely sketching it. Consistent with our presumption from the previous experiments, we found that participants reported a curved path in the facing perception in the articulation-only and natural locomotion condition and a curved path opposite the facing direction in the translation-only condition. Together, these findings further suggest that participants' perception of the path is affected by biological motion and that they have a bias toward perceiving curvilinear paths when either translation or articulation of biological motion is presented in isolation.

## General discussion

We studied how participants perceive self-motion through a crowd of point-light walkers. This perceptual scenario is complex (Riddell & Lappe, 2018; Hülemeier & Lappe, 2020; Koerfer & Lappe, 2020) as it involves optic flow analysis (Gibson, 1950; Longuet-Higgins & Prazdny, 1980; Lappe & Rauschecker, 1993) and biological motion processing (Johansson, 1973).

One of the issues is determining any source of rotation, i.e., a viewpoint rotation or a movement along a curved path (Royden, 1994; Royden et al., 1994; Li & Warren, 2000; Li & Warren, 2004; Royden et al., 2006). Although the visual system can accurately estimate heading from an instantaneous flow field (Li et al., 2006), determining the source of rotation, if present, from the instantaneous flow field alone is not possible because it requires the temporal evolution of the flow field and depth information (van den Berg, 1992; Stone & Perrone, 1997; Li & Warren, 2000). Observers often mistake rotations of the viewpoint for movements along a curved path, as first reported by Royden (1994), resulting in the misperception of trajectory and heading (Bertin et al., 2000; Cheng & Li, 2012).

Perceiving self-motion through a crowd adds to the complexity of processing human motion (Johansson, 1973; Masselink & Lappe, 2015). Such human motion violates the scene rigidity (Longuet-Higgins & Prazdny, 1980) and induces noise into the flow pattern, resulting in heading biases (Riddell & Lappe, 2017; Riddell & Lappe, 2018; Riddell et al., 2019; Hülemeier & Lappe, 2020; Koerfer & Lappe, 2020). On the other hand, the articulation pattern of biological motion provides valid information about the direction and speed of the walker (Giese & Lappe, 2002; Masselink & Lappe, 2015), which could reduce this noise and compensate for the independent motion (Riddell & Lappe, 2018; Hülemeier & Lappe, 2020).

Prior research unveiled small heading errors for static or naturally moving crowds, but excessive errors when the individual components of biological motion are shown in isolation, as in our translation-only and articulation-only conditions (Riddell & Lappe, 2018; Hülemeier & Lappe, 2020; Koerner & Lappe, 2020). These heading biases displayed differences in magnitude and direction among individuals, indicating highly idiosyncratic data (Hülemeier & Lappe, 2020). Our present research focused on understanding the source of these heading biases (object motion as evidence of a translation or a rotation of the viewpoint). Participants assessed their perceived heading direction and path with self-motion always following a straight path.

Empirical evidence for curvature percepts, their association with large heading errors, and the influence of optic flow deliver a coherent concept for illusory path perception when object motion is interpreted as evidence of a translation or a rotation of the viewpoint. Importantly, this illusory path perception solely occurs when the components of biological motion are presented in isolation (translation-only or articulation-only conditions).

For the static and natural locomotion conditions, we found straight path percept combined with small heading errors, aligning with our expectations. The static crowd forms a rigid environment enabling accurate self-motion perception from optic flow (Longuet-Higgins & Prazdny, 1980). In natural locomotion, prior research also reported relatively small heading biases despite the perturbations in the flow field due to the biological motion (Riddell & Lappe, 2018; Hülemeier & Lappe, 2020). Because heading was accurate in natural locomotion, we did not presume a misperception of the traveled path.

For the translation-only and articulation-only conditions, the results were also in line with our expectations. We observed significant curvilinear path percepts in the opposite directions in the translation-only and articulation-only conditions, accompanied by large heading biases (Riddell & Lappe, 2018; Hülemeier & Lappe, 2020). This misperception persisted in translation-only despite depth information and independent optic flow from the ground. In the articulation-only, the ground provided sufficient information to resolve the self-motion misperception, resulting in a straight path. When comparing the curvature direction, we observed that it was opposite to the walkers' motion in the translation-only condition, but aligned with the walker's facing direction in the articulation-only condition. The findings lend support that observers on average perceived the walker motion as evidence for self-rotation (Duffy & Wurtz, 1993; Li et al., 2018) in both conditions (Figure 1 lower row). However, consistent with earlier observations we found a large spread of heading directions and curvature across individuals, indicating again a high idiosyncrasy in the percepts. Thus, while on average the findings are consistent with the lower row expectations of Figure 1, quite a sizable number of participants reported percepts consistent with the upper row expectations of Figure 1. These participants perceived linear self-motion with heading biases in opposite walker-facing direction in the translation-only condition (Figure 1A), and in walker-facing direction in the articulation-only condition (Figure 1C) throughout the experiment (Figure 5). Similar idiosyncrasies were also seen in Experiment 2. Thus, on average, participants perceived walker motion as indicative of self-rotation, but for some, it was interpreted as evidence of self-translation (Li et al., 2018; Hülemeier & Lappe, 2020), leading to heading biases in opposite directions.

The crucial finding substantiating the illusory path perception as evidence for either self-rotation or self-translation is the relation between heading bias and perceived curvature. Participants exhibit heading bias and curvature estimates in opposite directions. This negative correlation supports the proposed link between path perception and heading estimation while accounting for the idiosyncratic differences in path and heading perception we observe at the individual level.

In the articulation-only condition, limb articulation induces heading biases by affecting the reference frame of self-motion encoding (Tadin, Lappin, Blake, & Grossman, 2002). Humans derive translation velocity from limb articulation (Masselink & Lappe, 2015). The perceived translation evokes the impression of a moving background, leading to illusory background motion (Fujimoto, 2003; Fujimoto & Sato, 2006; Fujimoto & Yagi, 2008). In combination with the forward translation of the observer, this illusory background motion could be perceived as self-rotation, producing heading misperception and the impression of moving along a curve (Li & Warren, 2000; Hülemeier & Lappe, 2020). When more information about the true self-motion was available (e.g., because of a visible ground), the perceived path became straight, and the heading error disappeared.

In the translation-only condition, perceived curvature may derive from difficulties in disentangling self-translational and self-rotational components of the observer's retinal flow (Royden, 1994, Royden et al. 1994; Li & Warren, 2000; Li & Warren, 2004; Royden et al., 2006). The instantaneous flow field in scenes with translating walkers is ambiguous as both linear translation and self-rotation could explain the origin of motion in the scene (Li et al., 2018; Hülemeier & Lappe, 2020). On average, it evokes an illusionary self-rotation leading to perceived curvature estimates in the opposite direction to the walker-facing direction.

In conclusion, we found that the knowledge about biological motion combined with the interpretation of object motion as evidence of self-translation or self-rotation within the flow field (Li et al., 2018) drives self-motion perception biases when only parts of biological motion are displayed. This bias leads to heading errors and misperceptions of the traveled path. Independent optic flow can partly resolve this bias.
